# *ISG15* mRNA transcript level in circulating leucocytes prognostic of overall survival in hepatocellular carcinoma patients and correlated with quality of life disturbances involved in anorexia-cachexia

**DOI:** 10.3389/fonc.2025.1589053

**Published:** 2025-08-27

**Authors:** Leung Li, Nelson L. S. Tang, Frankie Mo, Jane Koh, Edwin P. Hui, Brigette Ma, Stephen L. Chan, Kit F. Lee, Simon C. H. Yu, Winnie Yeo

**Affiliations:** ^1^ State Key Laboratory of Translational Oncology, Department of Clinical Oncology, Prince of Wales Hospital, Hong Kong Cancer Institute, Faculty of Medicine, The Chinese University of Hong Kong, Hong Kong, Hong Kong SAR, China; ^2^ State Key Laboratory of Translational Oncology, Department of Chemical Pathology, Prince of Wales Hospital, Li Ka Shing Institute of Health Sciences, Faculty of Medicine, The Chinese University of Hong Kong, Hong Kong, Hong Kong SAR, China; ^3^ Department of Surgery, Prince of Wales Hospital, Shatin, Faculty of Medicine, The Chinese University of Hong Kong, Hong Kong, Hong Kong SAR, China; ^4^ Department of Diagnostic and Interventional Radiology, Prince of Wales Hospital, Faculty of Medicine, The Chinese University of Hong Kong, Hong Kong, Hong Kong SAR, China

**Keywords:** type I interferon response, anorexia cachexia syndrome, liver cancer, interferon stimulated gene 15, qPCR, cDNA, EORTC QLQ-C30, HCC18 index score

## Abstract

**Background:**

An increasing number of immuno-therapeutic agents have proven efficacy in hepatocellular carcinoma (HCC). Inflammatory markers (c-reactive protein, interleukin-8 and inflammatory score) have been found to be prognostic factors in HCC patients. These inflammatory markers have demonstrated correlations with quality of life (QOL) disturbances in fatigue, appetite loss and nutritional concern. Type I interferon response triggered by HCC could be responsible for an inflammatory state and anorexia-cachexia syndrome leading to these specific QOL impairment. Peripheral blood Interferon Stimulated Gene 15 (*ISG15*) messenger ribonucleic acid (mRNA) transcript level, a biomarker for type I interferon response, was evaluated for its prognostic significance for overall survival (OS) in a prospective cohort of HCC patients. QOL measurement was employed to systemically capture and quantify patients’ clinical manifestations for correlations with *ISG15* mRNA transcript level.

**Methods:**

Clinical, QOL and laboratory data of 340 treatment naïve HCC patients were collected at study entry. *ISG15* mRNA transcript levels in circulating leucocytes were quantified. Independent prognostic factors for OS were identified. Correlation analyses between *ISG15* mRNA transcript level and scores of QOL factors were performed.

**Results:**

High *ISG15* mRNA transcript level in circulating leucocytes was an independent prognostic factor for poor OS (hazard ratio 1.62 [1.23-2.15]; p-value<0.01). The median OS of patients with high *ISG15* gene expression was significantly shorter than those with low expression, 4.7 versus 14.3 months respectively (p-value<0.03). There were significant correlations between high *ISG15* mRNA transcript level and worse scores in QLQ-C30 fatigue, appetite loss and QLQ-HCC18 nutritional disturbances (p-values <0.05).

**Conclusions:**

Elevated *ISG15* mRNA transcript level in peripheral blood leucocytes was an independent poor prognostic factor for OS in HCC patients. Patients with higher *ISG15* gene expression, suggesting more intense type I interferon response, had significantly worse OS. High *ISG15* gene expression demonstrated significant correlations with QOL disturbances in fatigue, appetite loss and nutritional concern. These QOL factors could be capturing the anorexia-cachexia manifestations from interferon response induced by HCC.

## 1 Introduction

Hepatocellular carcinoma (HCC) is the 3^rd^ leading cause of cancer death worldwide (1). Over one-half of HCC occur in China (2). More than 70% of HCC patients present with advanced disease and could only receive palliative or supportive therapy (3, 4). Prognostication therefore plays an important role, whereas two groups of prognostic factors have been gaining recognition: inflammatory markers and quality of life (QOL).

Immune-response and inflammation are closely related to HCC pathogenesis and progression (5). Inflammatory markers such as interleukin-8 (IL-8), inflammatory score (6) and c-reactive protein (CRP) (7) have been reported to be prognostic for overall survival (OS) in HCC patients. QOL assessment in HCC patients using the European Organization for Research and Treatment of Cancer (EORTC) QLQ-C30 and QLQ-HCC18 (8) also carried prognostic value.

Traditionally QOL disturbances in cancer patients were thought to be mostly related to mass effect from tumors. However, for uncertain reasons, QOL factors measuring appetite loss, nutritional disturbances, fatigue and functional impairment have been demonstrated to correlate with inflammatory markers (6, 9) in HCC patients. These QOL disturbances might involve a complex interplay between HCC and inflammatory cascade. IL-8, among these inflammatory markers, is a chemokine induced by type I interferon response (10). Interferon-α administration to mice, triggering type I interferon response, can lead to reduction in oral intake and wasting (11). In human studies, therapeutic use of interferon-α frequently caused adverse effects including anorexia, nausea, fatigue and asthenia in cancer as well as non-cancer patients (12–14). In other words, the above mentioned specific QOL factors appeared to be measuring the symptoms from type I interferon response. Therefore we postulated that type I interferon response could be activated in HCC patients, have prognostic significance and have association with QOL disturbances.

Plasma level of interferon could reflect interferon response, however, its measurement has been technically challenging with limited accuracy due to its low concentration with short half-life (15). On the other hand, Interferon Stimulated Gene 15 (*ISG15*) expression, reflecting type I interferon response activation, could be measured with higher accuracy (10, 16–20). Both endogenous type I interferon response or exogenous administration of type I interferon can lead to *ISG15* expression and enhanced IL8 production (17, 18). *ISG15* encodes for interferon-stimulated protein of 15 kilodalton, which is responsible for ISGylation of various targets to regulate immunologic functions (21). Specifically, *ISG15* expression was upregulated in viral infections including Coronarvirus Disease-2019 (COVID-19) (22–24), autoimmune diseases (25–27) and in some types of cancer (28–33). *ISG15* has demonstrated tumor-suppressing and tumor-promoting effects in different tumor types (30). For instance, high *ISG15* expression has been associated with progression of endometrial cancer (31) and earlier relapse in patients with resected HCC (32). On the contrary, *ISG15* expression has been implicated to suppress adenocarcinoma of lung (33) and cervical squamous cell carcinoma (34). While reported studies focused on intra-tumoral *ISG15* expression, there has been evidence to support that disease-associated interferon response gene expressions also manifested in circulating leucocytes (35). Hence we utilized *ISG15* mRNA level in circulating leucocytes to gauge the degree of type I interferon response (10, 16, 35).

In this study, we investigated the prognostic significance of *ISG15* mRNA transcript levels in peripheral blood leucocytes, clinical and QOL factors in HCC patients. QOL measurement was employed to systemically capture and quantify patients’ symptoms for statistical analyses and clinical correlation. The associations between *ISG15* mRNA transcript levels and QOL factors were evaluated.

## 2 Materials and methods

### 2.1 Patients

This study was approved by the Joint Chinese University of Hong Kong–New Territories East Cluster Clinical Research Ethics Committee, Prince of Wales Hospital (PWH), Hong Kong. Patients presented to PWH with newly diagnosed HCC were recruited prospectively. HCC diagnosis was established by either (i) the finding of hyper-vascular hepatic tumor(s) in dynamic imaging with elevated alpha-fetoprotein (AFP) or (ii) tumor biopsy. Patients were excluded from the study if they received prior HCC treatment, had a history of another malignancy, evidence of hepatic encephalopathy, cognitive impairment or illiteracy. All patients provided written informed consent for the study.

### 2.2 Data collection

Demographic, clinical, QOL data and peripheral blood were collected at study entry. After consenting, all patients had venesection for blood collection for once before HCC treatment. Blood was tested for *ISG15* mRNA transcript level, cell counts, renal and liver functions, clotting profile, AFP, hepatitis B surface-antigen and anti-hepatitis C antibody. Patients were followed-up until death.

### 2.3 Laboratory procedures for quantification of peripheral blood *ISG15* mRNA transcript level

#### 2.3.1 Peripheral blood leukocytes isolation and total RNA extraction

For *ISG15* gene expression analysis, peripheral blood in ethylenediaminetetraacetic acid (EDTA) was used. Density gradient centrifugation, by Lymphoprep™ (Serumwerk Bernburg AG, Germany), was used to isolate peripheral blood mononuclear cells (PBMC) and granulocytes. Extraction of total RNA from whole blood and leukocytes was performed using a modified acid guanidinium thiocyanate-phenol-chloroform (AGPC) protocol (36), according to the manufacturer’s instructions (Molecular Research Center, Inc., Ohio, USA). This process effectively isolated nucleated cells, the resulting total RNA yield would overwhelmingly be derived from leukocytes, with negligible contribution from anucleated erythrocytes and platelets. 250 µL TRI Reagent^®^ LS (TB126) was added to the PBMC and granulocytes and 300 µL TRI Reagent^®^ BD (TB120) was added to the whole blood. Phase separation was performed using 1-bromo-3-chloropropane (B9673, Sigma-Aldrich). Subsequent washing and elution were by isopropanol (I9516, Sigma-Aldrich) and ethanol (4116-4104, Daejung).

#### 2.3.2 Reverse transcription

Synthesis of first strand complementary deoxyribonucleic acid (cDNA) from the total RNA was performed using the PrimeScript RT Reagent Kit (#RR037A, Takara Bio, Shiga, Japan). 10 µL reverse transcription system was used, including 2 µL of 5X PrimeScript Buffer (for Real-Time), 0.5 µL PrimeScript RT Enzyme Mix I, 0.5 µL Oligo dT Primer (50 µM), 2 µL Random-6mers (100 µM), and 5 µL of RNA (Up to 1 µg/µL) from the samples. Reverse transcription was performed in the C1000 Touch Thermal Cycler (Bio-Rad, Hercules, California, USA), under these conditions: incubation at 37°C for 15 minutes, then enzyme denaturation at 85°C for 5 seconds, followed by storage at 4°C for short-term preservation before refrigeration storage.

#### 2.3.3 Quantification of *ISG15* gene expression

The cDNA was diluted and duplicated for optimization of the real time quantitative polymerase chain reaction (RT-qPCR) performance. In each RT-qPCR batch, the same reference sample was used and a serial 2-fold dilution was performed to establish PCR efficiency. The TB Green^®^ Premix Ex Taq™ II (Tli RNase H Plus) kit (#RR820A, Takara Bio, Shiga, Japan) and *ISG15* gene primers (see **Table 1**) were used in the reaction, carried out in LC480 thermal cycler (Roche, Basel, Switzerland). The qPCR conditions were: pre-incubation at 95°C for 30 seconds, then 45 cycles of amplification (95°C for 5 seconds, 55°C for 30 seconds, and 72°C for 20 seconds), melting (95°C for 1 minute, 40°C for 1 minute and 65°C for 20 seconds) and final cooling at 40°C for 30 seconds. Quantification of the expression level of the patients’ *ISG15* gene was by the efficiency (eff) corrected delta delta cycle threshold (ddCT) method. Gene *UBC* was used as the internal housekeeping gene. A constant cDNA sample from one healthy donor was used as the calibrator for all the tests. The formula for fold change (FC) calculation is shown in **Table 2**. All qPCR were performed in duplicate and the duplicate cycle threshold (Ct) value difference must be less than 1 for quality control (36–38).

**Table 1:** Primer sequences for quantification of peripheral blood *ISG15* gene expression.

Primer	Sequence
*ISG15* gene (forward)	5’- GGACCCAGCCACTCATCTTTGCCAGTACAGG - 3’
*ISG15* gene (reverse)	5’- CGACCGACCGCAGCTCTGACACCGACATG - 3’
Ubiquitin C gene (forward)	5’- CGTCCGTCGCCAGCCGGGATTTGGGTCG - 3’
Ubiquitin C gene (reverse)	5’- CGTCCGTCGCCAGCCGGGATTTGGGTCG - 3’

*ISG15* – interferon stimulated gene 15.

**Table 2:** Fold-change calculation for patients’ peripheral blood *ISG15* gene expression.

FC=eff *ISG15*^(Ct*ISG15*_calibrator - Ct*ISG15*_mean) ÷eff *UBC* ^(Ct*UBC*_calibrator - Ct*UBC*_mean)

Ct, cycle threshold; FC, fold change; *ISG15*, interferon stimulated gene 15; UBC, ubiquitin C gene.

### 2.4 QOL measurement

The EORTC QLQ-C30 (39) and QLQ-HCC18 (40) questionnaires were completed by patients at study entrance. C30 and HCC18 index scores, summary scorings of QLQ-C30 and QLQ-HCC18 respectively, were calculated (see **Supplementary Table 1**) (8).

### 2.5 Estimation of sample size and statistical analyses

The median OS of local HCC patients was previously reported to be 6 months (41). We assumed a hazard ratio (HR) of 2.0 for high *ISG15* gene expression, with two-sided alpha level at 0.05 and power at 80%, the estimated target sample size was 298 patients. Complete-case analysis was adopted since index scores calculation required all scores from QLQ-C30 and QLQ-HCC18 to be available and there has been no prior knowledge to allow logical imputation for missing data in a novel variable of peripheral blood *ISG15* mRNA transcript level. Allowing for 10% of patients with missing data, we aimed to recruit 331 patients. Clinical and QOL data were assessed by standard descriptive tests. OS was defined as the duration between study-entry and death. Time-to-death analysis was performed using the Kaplan-Meier method, censoring patients alive or lost to follow-up. Survival distributions were compared using the log-rank test. Prognostic clinical and QOL factors for OS were identified using Cox regressions. Significant non-overlapping clinical factors and QOL factors were analyzed in multivariate Cox regression models to adjust for confounding variables in order to identify independent prognostic factors. Two-tailed p-values of less than 0.05 were considered statistically significant. Receiver operating characteristic (ROC) curve of *ISG15* mRNA transcript level thresholds using leucocytosis as a surrogate outcome was generated to determine an optimal cut-off for high *ISG15* gene expression for correlation tests. Correlations between *ISG15* mRNA transcript level and QOL factors were analyzed using Student’s t-test, Mann-Whitney U test (since the novel variable of *ISG15* mRNA level had unknown distribution, both parametric and non-parametric tests were performed as sensitivity analyses), univariate and multivariate logistic regressions. P-values of less than 0.05 were considered significant. Analyses were performed using SAS version 9.4 (SAS institute, Cary, NC, USA).

## 3 Results

### 3.1 Patient characteristics

From 2009 to 2017, 340 HCC patients were recruited (318 patients with complete data for analyses, 20 with incomplete QOL data and 2 with unsuccessful *ISG15* mRNA quantification). Patients’ baseline characteristics, clinical and laboratory data are presented in **Table 3**. The median age at presentation was 59 (range 27 to 86) years. Most patients (87%) were male. Concerning the pattern of liver tumors: 32% of patients had uni-nodular disease, 28% had multi-nodular disease and 40% had diffuse-infiltrative disease. Portal-vein thrombosis was found in 98 (31%) patients, while metastasis beyond liver in 72 (23%) patients. Evidence of hepatitis B was found in 80% of patients. Two-hundred and thirty-one (73%) patients were in Child-Pugh class A, 75 (24%) in class B and 11 (4%) in class C. The median follow-up duration was 116 (95% confidence interval [CI] 87-120) months. The median OS was 9.5 (95% CI 6.1-14.3) months.

**Table 3:** Clinical and laboratory characteristics of the patients.

Clinical factor	N	Percentage	Hazard Ratio	95% Confidence Intervals	P-value
Age ≤65 year	220	69.2	0.901	0.697-1.164	0.4239
Male	278	87.4	1.189	0.825-1.713	0.3526
ECOG ≥2	17	5.4	3.894	2.355-6.439	<0.0001
HBsAg+	254	79.9	1.014	0.758-1.357	0.9235
Anti-HCV+	25	7.9	1.138	0.753-1.719	0.5597
Ascites	76	23.9	2.599	1.979-3.414	<0.0001
Cirrhosis	157	49.4	0.801	0.630-1.018	0.0699
Child-Pugh					
Class A	231	72.6	1	–	–
Class B	75	23.9	2.2	1.670-2.900	<0.0001
Class C	11	3.5	10.322	5.429-19.623	<0.0001
AFP ≥200ng/mL	147	46.2	2.227	1.744-2.843	<0.0001
Hb ≤10g/dL	19	6	2.127	1.330-3.402	0.0016
WCC >10x10^9/L	40	12.6	2.869	2.024-4.066	<0.0001
Platelet count <100x10^9/L	48	15.1	0.648	0.461-0.912	0.0129
INR >1.4	14	4.4	1.66	0.930-2.965	0.0866
Creatinine ≥ULN	44	13.8	1.243	0.888-1.740	0.2055
Bilirubin ≥ 20umol/L	141	44.3	1.962	1.541-2.498	<0.0001
Albumin ≤35 g/l	105	33	2.357	1.830-3.035	<0.0001
ALT >2xULN	48	15.1	1.616	1.161-2.251	0.0045
ALP >2xULN	87	27.4	2.609	2.002-3.401	<0.0001
*ISG15* mRNA fold change >2.93	79	24.8	1.367	1.038-1.798	0.0258
Tumor morphology					
Uni-nodular	102	32.1	1		
Multi-nodular	90	28.3	1.289	0.991-1.675	0.0581
Diffuse	126	39.6	3.306	2.553-4.282	<0.0001
Extrahepatic metastasis	72	22.6	4.552	3.361-6.164	<0.0001
Portal vein thrombosis	98	30.8	3.161	2.418-4.132	<0.0001
AJCC stage			2.46	2.112-2.866	<0.0001
I or II	108	34	1	–	–
III	39	12.3	2.383	1.569-3.621	<0.0001
IV	171	53.7	6.033	4.437-8.202	<0.0001
BCLC stage			2.418	2.063-2.835	<0.0001
A	58	18.2	1	–	–
B	60	18.9	3.21	2.033-5.071	<0.0001
C	162	50.9	5.461	3.634-8.208	<0.0001
D	38	11.9	19.978	11.870-33.626	<0.0001
First line treatment					
Curative	84	26.4	1	–	–
Palliative or best supportive care	234	73.6	6.075	4.328-8.529	<0.0001

AFP, α-fetoprotein; AJCC, the American Joint Committee on Cancer staging system; ALP, alkaline phosphatase; ALT, alanine aminotransferase; anti-HCV+, anti-hepatitis C antibody positive; BCLC, the Barcelona Clinic Liver Cancer staging system; ECOG, Eastern Cooperative Oncology Group performance status; Hb, hemoglobin; HBsAg+, hepatitis B surface antigen positive; INR, international normalized ratio; *ISG15*, interferon stimulated gene 15; n, number; ULN, upper limit of normal; mRNA messenger ribonucleic acid; WCC, white cell count.

**Table 4** presents the QOL data of the patients. The mean QLQ-C30 physical functioning score was 74 (standard deviation [SD] 23), fatigue score was 43 (SD 30), appetite loss score was 33 (SD 35), QLQ-HCC18 jaundice score was 22 (SD 20), nutritional concern score was 27 (SD 22) and pain score was 21 (SD 24).

**Table 4:** Patients’ quality of life parameters.

QOL factor	Mean	Standard Deviation	Hazard Ratio	95% Confidence Intervals		P-value
EORTC QLQ-C30						
Physical Functioning	73.77	23.48	0.369	0.291	0.468	<0.0001
Role Functioning	74.79	32.77	0.506	0.427	0.6	<0.0001
Emotional Functioning	71.2	23.5	0.745	0.569	0.975	0.0325
Cognitive Function	77.2	23.35	0.67	0.519	0.865	0.0021
Social Functioning	68.34	30.23	0.53	0.431	0.651	<0.0001
Global health status/QoL	53.62	25.17	0.432	0.334	0.559	<0.0001
Fatigue	42.66	29.91	2.299	1.886	2.802	<0.0001
Nausea and vomiting	12.26	21.83	1.779	1.39	2.277	<0.0001
Pain	30.24	30.62	2.043	1.686	2.476	<0.0001
Dyspnoea	29.35	31.3	1.739	1.434	2.11	<0.0001
Insomnia	43.82	36.04	1.48	1.247	1.757	<0.0001
Appetite loss	33.33	34.93	2.14	1.8	2.546	<0.0001
Constipation	14.99	24.6	1.19	0.939	1.509	0.1508
Diarrhea	18.24	27.57	1.466	1.168	1.84	0.001
Financial difficulties	51.36	37.02	1.29	1.095	1.521	0.0025
C30 index score	30.49	18.83	3.995	2.928	5.45	<0.0001
EORTC QLQ-HCC18						
Fatigue	33.89	25.19	2.468	1.943	3.135	<0.0001
Body Image	25.68	23.36	2.007	1.619	2.487	<0.0001
Jaundice	22.01	20.49	1.523	1.177	1.97	0.0014
Nutrition	27.37	22.08	2.835	2.15	3.737	<0.0001
Pain	21.44	23.77	2.069	1.611	2.656	<0.0001
Fever	5.71	13.68	1.785	1.162	2.742	0.0082
Sex life	26.31	32.47	1.217	1.004	1.475	0.0451
Abdominal swelling	29.14	33.96	2.043	1.708	2.444	<0.0001
HCC18 index score	23.94	16.53	3.775	2.716	5.248	<0.0001

EORTC, the European Organization for Research and Treatment; QOL, quality of life.

### 3.2 *ISG15* mRNA analyses

**Figure 1:**
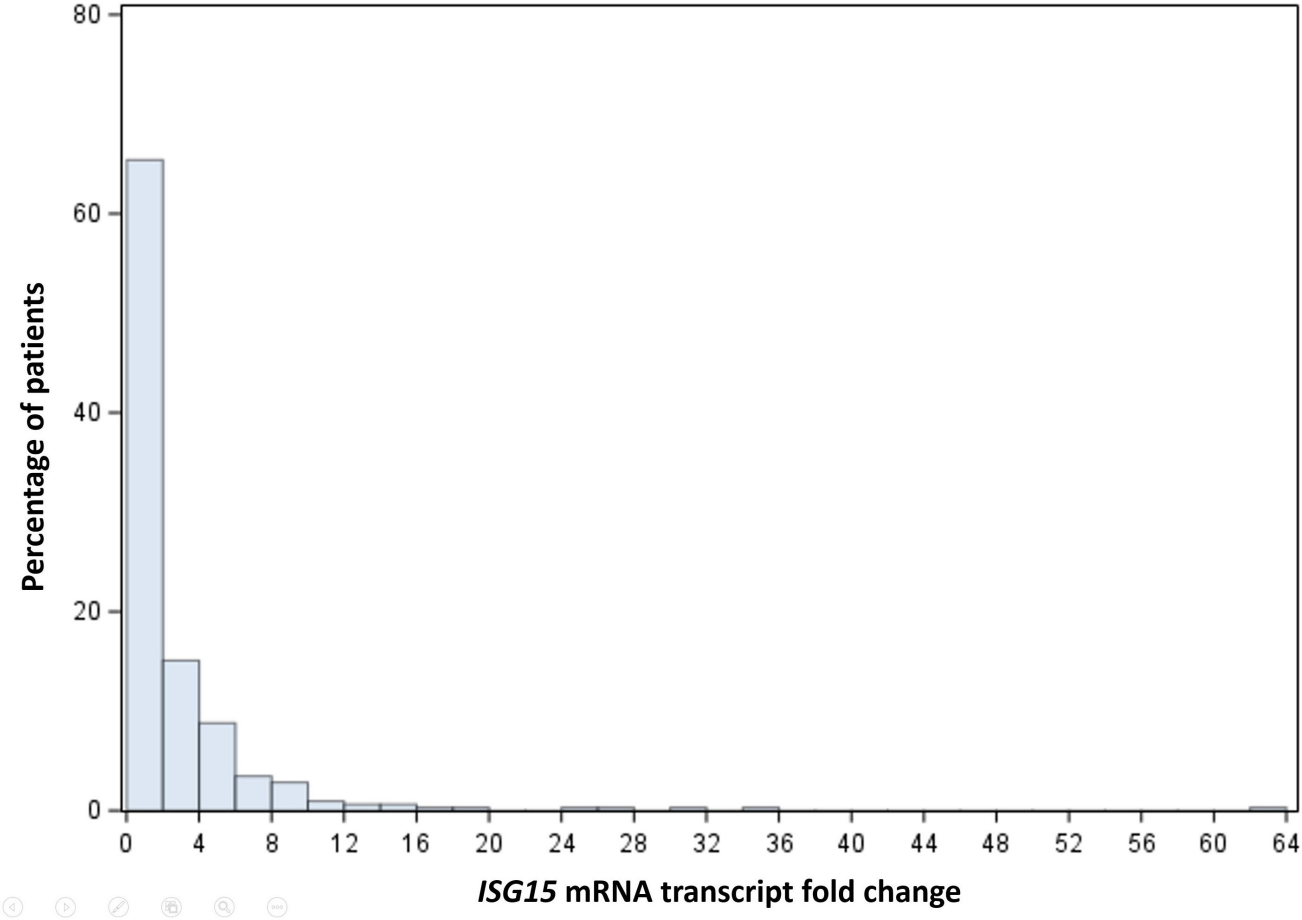
Histogram showing the distribution of *ISG15* mRNA transcript fold change in HCC patients.

**Figure 2:**
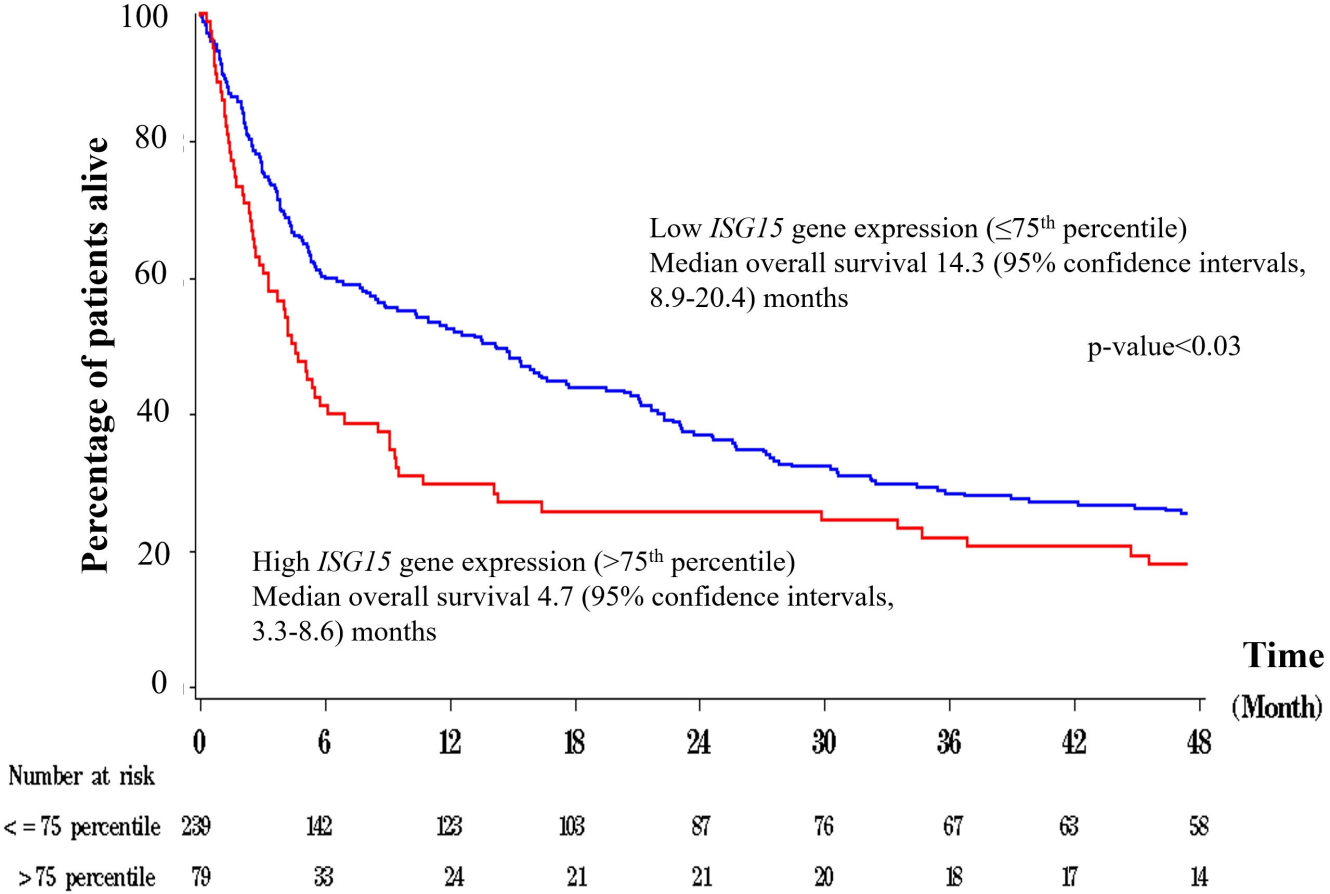
Overall survival plots of patients with low versus high peripheral blood *ISG15* gene expression.

The median peripheral blood *ISG15* mRNA transcript level was 1.24 (interquartile range 0.57-2.93) FC. The distribution of ISG15 mRNA transcript FC in HCC patients is shown in **Figure 1**. *ISG15* mRNA level exhibited a skewed distribution, therefore levels of more than 75^th^ percentile (2.93 FC) were grouped as high expression in Cox regressions for survival analyses. The median OS was significantly worse in patients with high *ISG15* gene expression (signifying a higher degree of type I interferon response) when compared to those with low expression, 4.7 (95% CI 3.3-8.6) versus 14.3 (95% CI 8.9-20.4) months respectively (log-rank p-value <0.03). **Figure 2** presents the survival curves of patients according to *ISG15* gene expression levels.

### 3.3 Univariate Cox analyses of *ISG15* mRNA level, clinical and QOL prognostic factors

Univariate Cox regressions of clinical and QOL factors are presented in **Tables 3**, **4** respectively. Patients with high *ISG15* mRNA level had significantly higher risk of death than those with low level (p<0.03). Compared to patients in Child-Pugh class A, patients in Child-Pugh’s B or C classes had significantly worse OS (p-values <0.01). Compared to patients with American Joint Committee on Cancer stage I and II diseases, patients with stage III or IV diseases had significantly worse OS (p-values <0.01). Raised white cell count (WCC), lower hemoglobin level and extrahepatic metastasis were also significantly associated with worse OS (p-values <0.01).

Better (higher) scores in QLQ-C30 physical functioning, role functioning, emotional functioning, cognitive functioning, social functioning and global health status/QoL were significantly associated with better OS (p-values <0.05). Worse (higher) scores in QLQ-C30 fatigue, nausea and vomiting, pain, dyspnea, insomnia, appetite loss, diarrhea, financial difficulties, QLQ-HCC18 fatigue, body image, jaundice, nutrition, pain, fever, sex life and abdominal swelling were significantly associated with worse OS (p-values <0.05). Worse (higher) C30 and HCC18 index scores were also significantly associated with worse OS (p-values <0.01).

### 3.4 Multivariate Cox analyses of prognostic factors

Multivariate Cox regressions using *ISG15* mRNA transcript level, clinical and QOL factors are presented in **Table 5**. Using QLQ-C30 as QOL factors, higher *ISG15* mRNA transcript level in circulating leucocytes (HR 1.62, 95% CI 1.23-2.15; p-value <0.01), higher white cell count (HR 1.09, 95% CI 1.05-1.14; p-value <0.01), the presence of extra-hepatic metastasis (HR 3.03, 95% CI 2.20-4.18; p-value <0.01), worse (higher) scores in appetite loss (HR 1.68, 95% CI 1.38-2.05; p-value <0.01) and C30 index score (HR 3.52, 95% CI 2.54-4.89; p-value <0.01) were independent prognostic factors for worse OS. Whereas higher hemoglobin level (HR 0.91, 95% CI 0.85-0.98; p-value <0.01) and better (higher) QLQ-C30 physical functioning score (HR 0.58, 95% CI 0.43-0.78; p-value <0.01) were independent prognostic factors for better OS.

**Table 5:** Multivariate Cox regressions of prognostic factors for overall survival.

Prognostic factors	EORTC Factors				Index Scores			
	Hazard Ratio	95% Confidence Intervals		P-value	Hazard Ratio	95% Confidence Intervals		P-value
QLQ-C30								
Physical Functioning	0.58	0.434	0.775	0.0002				
Appetite loss	1.68	1.375	2.051	<0.0001				
C30 index score					3.52	2.535	4.888	<0.0001
White cell count	1.094	1.054	1.137	<0.0001	1.099	1.058	1.141	<0.0001
Hemoglobin-level	0.912	0.853	0.976	0.0076	0.885	0.828	0.946	0.0003
Extra-hepatic metastasis	3.033	2.198	4.184	<0.0001	2.94	2.134	4.052	<0.0001
High *ISG15* gene expression	1.623	1.225	2.15	0.0007	1.626	1.228	2.154	0.0007
QLQ-HCC18								
Fatigue	1.428	1.015	2.011	0.0412				
Nutrition	1.523	1.028	2.257	0.0357				
Abdominal Swelling	1.352	1.091	1.676	0.0059				
HCC18 index score					3.353	2.373	4.74	<0.0001
White cell count	1.095	1.055	1.136	<0.0001	1.092	1.052	1.133	<0.0001
Hemoglobin-level	0.887	0.829	0.95	0.0005	0.878	0.821	0.939	0.0001
Extra-hepatic metastasis	3.013	2.183	4.159	<0.0001	3.17	2.302	4.365	<0.0001
High *ISG15* gene expression	1.48	1.121	1.954	0.0056	1.495	1.132	1.973	0.0046

EORTC, the European Organization for Research and Treatment; *ISG15*, interferon stimulated gene 15.

Using QLQ-HCC18 as QOL factors, higher *ISG15* mRNA transcript level (HR 1.48, 95% CI 1.12-1.95; p-value <0.01), presence of extra-hepatic metastasis (HR 3.01, 95% CI 2.18-4.16; p-value <0.01), higher white cell count (HR 1.10, 95% CI 1.06-1.14; p-value <0.01), worse (higher) scores in fatigue (HR 1.43, 95% CI 1.02-2.01; p-value <0.05), nutrition (HR 1.52, 95% CI 1.03-2.26; p-value <0.05), abdominal swelling (HR 1.35, 95% CI 1.09-1.68; p-value <0.01) and HCC18 index score (HR 3.35, 95% CI 2.37-4.74; p-value <0.01) were independent prognostic factors for worse OS. Whereas higher hemoglobin level (HR 0.89, 95% CI 0.83-0.95; p-value <0.01) was an independent prognostic factor for better OS.

### 3.5 Correlations between *ISG15* mRNA transcript level and clinical symptomatology

Correlation analyses between *ISG15* mRNA transcript level and QOL factors are presented in **Table 6**. From ROC analysis, the optimal threshold for high *ISG15* mRNA transcript level for correlation analyses was 1.3 FC or above. Worse (higher) scores in QLQ-C30 fatigue (T-test p-value <0.05; Mann-Whitney U p-value <0.05; logistic regression odds ratio [OR] 1.51, 95% CI 1.002-2.270; p-value <0.05), appetite loss (T-test p-value <0.05; Mann-Whitney U p-value <0.03; OR 1.48, 95% CI 1.03-2.12; p-value <0.05) and QLQ-HCC18 nutritional concern (T-test p-value <0.05; Mann-Whitney U p-value <0.03; OR 1.83, 95% CI 1.03-3.26; p-value <0.05) demonstrated significant correlations with higher *ISG15* mRNA transcript level.

**Table 6:** Correlation analyses between *ISG15* gene expression and quality of life factors.

QOL factors	Low *ISG15* expression	High *ISG15* expression	Student’s T-test	Mann-Whitney U test	Logistic regression	
	Mean (SD)	Mean (SD)	P-value	P-value	Odds ratio (95% CI)	P-value
QLQ-C30						
Physical Functioning	74.8 (23.7)	73.3 (22.8)	0.601	0.4835	0.87 (0.52-1.46)	0.5996
Role Functioning	77.5 (32.0)	73.8 (33.5)	0.3519	0.2617	0.84 (0.58-1.21)	0.3508
Emotional Functioning	71.4 (21.4)	72.0 (25.4)	0.8424	0.4543	1.052 (0.64-1.74)	0.8444
Cognitive Function	77.4 (20.3)	77.6 (25.2)	0.9245	0.3937	1.025 (0.61-1.72)	0.9258
Social Functioning	70.6 (29.8)	67.3 (31.2)	0.3782	0.3971	0.838 (0.57-1.24)	0.3769
Global Health Status/QoL	54.3 (25.1)	54.9 (25.6)	0.8288	0.9295	1.053 (0.66-1.69)	0.828
Fatigue	37.4 (29.8)	44.6 (29.5)	0.0475	0.0365	1.51 (1.002-2.27)	0.0485
Nausea and vomiting	10.6 (20.4)	12.7 (22.2)	0.4081	0.5376	1.27 (0.72-2.24)	0.4077
Pain	28.3 (29.0)	28.2 (30.0)	0.9689	0.8381	1.00 (0.66-1.48)	0.9688
Dyspnoea	28.2 (30.8)	29.8 (31.4)	0.6645	0.6745	1.09 (0,74-1.60)	0.6632
Insomnia	38.5 (33.1)	44.3 (37.2)	0.1765	0.2271	1.26 (0.90-1.78)	0.1761
Appetite loss	26.6 (32.8)	35.5 (35.1)	0.0309	0.0243	1.48 (1.03-2.12)	0.032
Constipation	13.8 (24.9)	15.8 (24.9)	0.5143	0.3888	1.18 (0.72-1.91)	0.513
Diarrhea	16.0 (25.0)	19.7 (29.6)	0.2642	0.455	1.29 (0.83-2.00)	0.2641
Financial difficulties	47.7 (37.5)	53.5 (37.0)	0.199	0.1998	1.23 (0.90-1.70)	0.1984
C30 Index score	28.1 (18.3)	31.0 (18.8)	0.1929	0.1539	1.54 (0.81-2.95)	0.1927
QLQ-HCC18						
Fatigue	31.4 (25.7)	35.1 (24.7)	0.221	0.1645	1.35 (0.84-2.18)	0.2208
Body Image	23.6 (24.9)	26.3 (21.2)	0.3248	0.0985	1.30 (0.77-2.21)	0.3242
Jaundice	20.2 (19.9)	22.9 (20.5)	0.2673	0.2356	1.40 (0.77-2.55)	0.2671
Nutrition	23.7 (21.0)	29.2 (21.6)	0.037	0.0213	1.83 (1.03-3.26)	0.0385
Pain	19.4 (22.9)	21.2 (24.3)	0.5352	0.6155	1.18 (0.71-1.95)	0.5339
Fever	5.8 (14.2)	6.0 (14.2)	0.9055	0.8971	1.05 (0.45-2.45)	0.9053
Sex life	25.5 (30.2)	27.6 (34.5)	0.5862	0.8768	1.11 (0.77-1.60)	0.5848
Abdominal swelling	26.3 (32.6)	32.0 (34.9)	0.1645	0.183	1.29 (0.90-1.84)	0.1645
HCC18 index score	22.0 (16.3)	25.0 (16.7)	0.128	0.0952	1.77 (0.85-3.68)	0.1287

CI, confidence intervals; QOL, quality of life; *ISG15*, interferon stimulated gene 15; SD, standard deviation.

Results of multivariate logistic regressions between *ISG15* mRNA transcript level and QOL factors adjusting for clinical factors are presented in **Supplementary Table 2**. Factors that best predict a higher peripheral blood *ISG15* mRNA transcript level were worse (higher) scores in QLQ-C30 appetite loss (OR 1.48, 95% CI 1.03-2.12; p-value <0.05) and QLQ-HCC18 nutritional concern (OR 1.83, 95% CI 1.03-3.26; p-value <0.05).

## 4 Discussion

This is the first study to show peripheral blood *ISG15* mRNA transcript level as an independent prognostic factor for OS in HCC patients. Patients with high level of *ISG15* mRNA in circulating leucocytes compared to patients with low level had significantly worse survival, with median OS of 4.7 and 14.3 months respectively. In contrast to a previous report which has shown prognostic value of intra-tumoral ISG15 protein expression confining to early stage HCC patients who received surgical resection, the current study using peripheral blood *ISG15* mRNA transcript level demonstrated prognostic significance applicable to all stages of HCC. Moreover, measuring *ISG15* mRNA transcript level in peripheral blood (commonly referred to as liquid biopsy) has the advantage of being more practical than measuring ISG15 expression in tumor-tissue. Since majority of HCC are diagnosed by imaging findings with tumor markers and risk factors evaluation (42), tumor biopsy is usually unavailable for testing.

This is also the first study to report *ISG15* mRNA level in circulating leucocytes as an independent prognostic factor in the cancer field. Since some other cancers have demonstrated ISG15 protein overexpression in tumor-tissue, further studies are warranted to investigate whether peripheral blood *ISG15* mRNA level is also prognostic in other cancer types.

As part of the survival analyses, QOL has demonstrated significant prognostic value. Patients with worse baseline scores in physical functioning, fatigue, appetite loss, nutritional disturbance and abdominal swelling had worse survival compared to those with better scores. QLQ-C30 appetite loss, QLQ-HCC18 nutritional disturbances and abdominal swelling were independent prognostic factors for OS that were not previously reported.

Apart from viral infection, cancer has also been implicated to activate interferon response (43–45). Since *ISG15* gene expression has been reported to be specific for type I interferon response pathway activation (16), the current results of elevated *ISG15* expression in treatment-naïve HCC patients suggested that type I interferon response was involved in HCC. All recruited patients were treatment-naïve, therefore they did not have any confounding inflammatory response from HCC therapy.

QOL measurement systematically assesses common symptoms and functional disturbances in HCC patients and provides quantification of severity of symptoms in ratings. These data allow correlation analyses between intensity of interferon response pathway activation (as reflected by *ISG15* mRNA transcript level) and clinical symptomatology.

*ISG15* mRNA level was correlated with HCC patients’ symptomatology. In particular, QOL factors (fatigue. appetite loss and nutrition [which includes questioning for patient’s worries about poor nourishment and recent weight loss]) demonstrated significant correlations with *ISG15* gene expression, a pattern consistent with results from analyses using IL-8, CRP and inflammatory score (6, 7). This is logical, since fatigue and anorexia are common clinical manifestations after interferon-α injection (triggering type I interferon response). The cytokines released from type I interferon response have been implicated in inducing anorexia in the central nervous system level, causing reduced oral intake and wasting in cancer patients (the cancer anorexia-cachexia syndrome) (46). The current data suggested that these QOL disturbances have strong underlying biological basis related to interferon response.

In other words, QOL disturbances involving fatigue, appetite loss and nutrition altogether could suggest type I interferon response triggered by HCC. Such grouping of QOL factors could be utilized as a biomarker itself. Future studies could evaluate whether transforming these QOL item scores into an aggregate-score could reflect the intensity of type I interferon response.

Since these QOL impairment could be due to interferon response activation, controlling interferon response might relieve HCC patients’ symptoms and thereby improve their QOL. HCC control with anti-cancer therapy might reduce the intensity of type I interferon response. Furthermore, clinical studies suggested that adverse effects from therapeutic interferon-α could be treated with various measures, e.g. methylphenidate and corticosteroid for fatigue; megestrol acetate and metoclopramide for anorexia; as well as dietary supplementation for nutritional disturbance (47). Further research is necessary to investigate the effectiveness of these measures in improving patients’ QOL.

In recent years, various immune checkpoint inhibitors including anti-programmed death-1 (anti-PD-1), anti-programmed death ligand-1 (anti-PD-L1) and anti-cytotoxic T-lymphocyte-associated antigen 4 (anti-CTLA-4) classes have proven efficacy in treatment for HCC (48–53). *ISG15*’s key involvement in immune defense system has gained research interest in developing it as a target for cancer immunotherapy. For instance, vaccine against ISG15 protein antigen, ISG15 gene knockdown, ISG15 targeting to potentiate anti-PD-1’s efficacy in pre-clinical cancer models showed some early promises (54–56). Since *ISG15* has demonstrated both tumor-suppressing as well as tumor-promoting properties even within the same tumor type (57, 58), complex mechanistic interplays between *ISG15* and other immunologic factors are likely involved in immune response against cancer cells. Further investigation is warranted to elucidate *ISG15*’s pivotal immune-regulatory function. Nevertheless, the current findings established the prognostic characteristics of *ISG15* gene expression in HCC patients. Peripheral blood *ISG15* mRNA transcript level is a candidate for evaluation as predictive biomarker in future HCC immunotherapy clinical trials. For instance, neutrophil-to-lymphocyte ratio (an inflammatory marker) has been suggested to be predictive of efficacy of immunotherapy in head and neck cancer (59).

We acknowledge there were several limitations in this study. First, our study focused on HCC patients, it is unknown whether the current findings related to ISG15 mRNA transcript level is generalizable to other malignant and non-malignant diseases. Second, in order to capture patient-reported QOL, patients with cognitive impairment were excluded. This prevented recruitment of some terminally-ill patients with encephalopathy due to hepatic failure. To minimize this potential selection-bias in future studies, we could include patients with cognitive impairment by accepting carer-proxy rated QOL data for analysis. Third, our study did not capture post-treatment ISG15 expression and QOL data, which could provide information regarding whether treatment response would alter patients’ ISG15 expression or QOL. However, the current findings pave the path for future research in this direction.

Our study possessed several strengths. First, the sample size was large enough to compensate for the number of missing cases (6.4%) while maintaining adequate power for statistical analyses. Second, this study had a median follow-up duration of 9 years which allow capturing of long survival data, thereby enhancing the precision of survival analyses.

## 5 Conclusion

This is the first study to demonstrate *ISG15* mRNA transcript level in peripheral blood leucocytes as an independent prognostic factor for OS in HCC patients. Patients with higher *ISG15* gene expression, reflecting more intense interferon response pathway activation, had significantly worse OS. High *ISG15* mRNA transcript level in circulating leucocytes was significantly correlated with QOL disturbances in terms of fatigue, appetite loss and nutritional concern in HCC patients. These QOL factors could be registering the anorexia-cachexia manifestations from type I interferon response triggered by HCC.

## Data Availability

The raw data supporting the conclusions of this article will be made available by the authors, without undue reservation.

## References

[1] Forner A Reig M Bruix J. Hepatocellular carcinoma. Lancet. (2018) 391:1301–14. doi: 10.1016/S0140-6736(18)30010-2, PMID: 29307467

[2] El-Serag HB Rudolph KL. Hepatocellular carcinoma: epidemiology and molecular carcinogenesis. Gastroenterology. (2007) 132:2557–76. doi: 10.1053/j.gastro.2007.04.061, PMID: 17570226

[3] Xiang X Zhong JH Wang YY You XM Ma L Xiang BD. Distribution of tumor stage and initial treatment modality in patients with primary hepatocellular carcinoma. Clin Trans oncology: Off Publ Fed Spanish Oncol Societies Natl Cancer Institute Mexico. (2017) 19:891–7. doi: 10.1007/s12094-017-1621-6, PMID: 28160206

[4] Siegel RL Giaquinto AN Jemal A. Cancer statistics, 2024. CA Cancer J Clin. (2024) 74:12–49. doi: 10.3322/caac.21820, PMID: 38230766

[5] Hernandez-Gea V Toffanin S Friedman SL Llovet JM. Role of the microenvironment in the pathogenesis and treatment of hepatocellular carcinoma. Gastroenterology. (2013) 144:512–27. doi: 10.1053/j.gastro.2013.01.002, PMID: 23313965 PMC3578068

[6] Li L Chan SL Mo F Hui EP Koh J Chan AKC. Correlations of health-related quality of life with serum inflammatory indicators IL-8 and mIBI in patients with hepatocellular carcinoma. Cancer Manag Res. (2019) 11:2719–27. doi: 10.2147/CMAR.S178482, PMID: 31040713 PMC6452825

[7] Kinoshita A Onoda H Imai N Iwaku A Oishi M Tanaka K. The C-reactive protein/albumin ratio, a novel inflammation-based prognostic score, predicts outcomes in patients with hepatocellular carcinoma. Ann Surg Oncol. (2015) 22:803–10. doi: 10.1245/s10434-014-4048-0, PMID: 25190127

[8] Li L Mo FK Chan SL Hui EP Tang NS Koh J. Prognostic values of EORTC QLQ-C30 and QLQ-HCC18 index-scores in patients with hepatocellular carcinoma - clinical application of health-related quality-of-life data. BMC cancer. (2017) 17:8. doi: 10.1186/s12885-016-2995-5, PMID: 28052758 PMC5209840

[9] Li L Chan SL Mo F Hui EP Koh J Chan AK. Status of inflammation in relation to health related quality of life in hepatocellular carcinoma patients. Qual Life Res. (2019) 28(9):2597–607. doi: 10.1007/s11136-019-02190-0, PMID: 31037590

[10] Lippitz BE. Cytokine patterns in patients with cancer: a systematic review. Lancet Oncol. (2013) 14:e218–28. doi: 10.1016/S1470-2045(12)70582-X, PMID: 23639322

[11] Stutte S Ruf J Kugler I Ishikawa-Ankerhold H Parzefall A Marconi P. Type I interferon mediated induction of somatostatin leads to suppression of ghrelin and appetite thereby promoting viral immunity in mice. Brain Behav Immun. (2021) 95:429–43. doi: 10.1016/j.bbi.2021.04.018, PMID: 33895286

[12] Motzer RJ Rakhit A Ginsberg M Rittweger K Vuky J Yu R. Phase I trial of 40-kd branched pegylated interferon alfa-2a for patients with advanced renal cell carcinoma. J Clin Oncol. (2001) 19:1312–9. doi: 10.1200/JCO.2001.19.5.1312, PMID: 11230473

[13] Smith D Wagstaff J Thatcher N Scarffe H. A phase I study of rDNA alpha-2b interferon as a 6-week continuous intravenous infusion. Cancer chemotherapy Pharmacol. (1987) 20:327–31. doi: 10.1007/BF00262586, PMID: 3690806

[14] Woo MH Burnakis TG. Interferon alfa in the treatment of chronic viral hepatitis B and C. Ann Pharmacother. (1997) 31:330–7. doi: 10.1177/106002809703100312, PMID: 9066942

[15] Rodero MP Decalf J Bondet V Hunt D Rice GI Werneke S. Detection of interferon alpha protein reveals differential levels and cellular sources in disease. J Exp Med. (2017) 214:1547–55. doi: 10.1084/jem.20161451, PMID: 28420733 PMC5413335

[16] Platanias LC. Mechanisms of type-I- and type-II-interferon-mediated signalling. Nat Rev Immunol. (2005) 5:375–86. doi: 10.1038/nri1604, PMID: 15864272

[17] Oh SJ Gim JA Lee JK Park H Shin OS. Coxsackievirus B3 infection of human neural progenitor cells results in distinct expression patterns of innate immune genes. Viruses. (2020) 12(3):325. doi: 10.3390/v12030325, PMID: 32192194 PMC7150933

[18] Serrano-Fernández P Möller S Goertsches R Fiedler H Koczan D Thiesen HJ. Time course transcriptomics of IFNB1b drug therapy in multiple sclerosis. Autoimmunity. (2010) 43:172–8. doi: 10.3109/08916930903219040, PMID: 19883335

[19] Tang FH Chang WA Tsai EM Tsai MJ Kuo PL. Investigating novel genes potentially involved in endometrial adenocarcinoma using next-generation sequencing and bioinformatic approaches. Int J Med Sci. (2019) 16:1338–48. doi: 10.7150/ijms.38219, PMID: 31692912 PMC6818189

[20] Yuan Y Ma H Ye Z Jing W Jiang Z. Interferon-stimulated gene 15 expression in systemic lupus erythematosus: Diagnostic value and association with lymphocytopenia. Z Rheumatol. (2018) 77:256–62. doi: 10.1007/s00393-017-0274-8, PMID: 28204879

[21] Wan XX Chen HC Khan MA Xu AH Yang FL Zhang YY. ISG15 inhibits IFN-α-resistant liver cancer cell growth. BioMed Res Int. (2013) 2013:570909. doi: 10.1155/2013/570909, PMID: 24024201 PMC3762208

[22] Skaug B Chen ZJ. Emerging role of ISG15 in antiviral immunity. Cell. (2010) 143:187–90. doi: 10.1016/j.cell.2010.09.033, PMID: 20946978 PMC2981609

[23] Teng TS Foo SS Simamarta D Lum FM Teo TH Lulla A. Viperin restricts chikungunya virus replication and pathology. J Clin Invest. (2012) 122:4447–60. doi: 10.1172/JCI63120, PMID: 23160199 PMC3533538

[24] Fassan M Collesei A Angerilli V Sbaraglia M Fortarezza F Pezzuto F. Multi-design differential expression profiling of COVID-19 lung autopsy specimens reveals significantly deregulated inflammatory pathways and SFTPC impaired transcription. Cells. (2022) 11(6):1011. doi: 10.3390/cells11061011, PMID: 35326463 PMC8947344

[25] Xiang M Chen Q Feng Y Wang Y Wang J Liang J. Bioinformatic analysis of key biomarkers and immune filtration of skin biopsy in discoid lupus erythematosus. Lupus. (2021) 30:807–17. doi: 10.1177/0961203321992434, PMID: 33530816

[26] Piera-Velazquez S Mendoza FA Addya S Pomante D Jimenez SA. Increased expression of interferon regulated and antiviral response genes in CD31+/CD102+ lung microvascular endothelial cells from systemic sclerosis patients with end-stage interstitial lung disease. Clin Exp Rheumatol. (2021) 39:1298–306. doi: 10.55563/clinexprheumatol/ret1kg, PMID: 33253099

[27] Yang Y Song J Zhao H Zhang H Guo M. Patients with dermatomyositis shared partially similar transcriptome signature with COVID-19 infection. Autoimmunity. (2023) 56:2220984. doi: 10.1080/08916934.2023.2220984, PMID: 37353938

[28] Jessen C Kreß JKC Baluapuri A Hufnagel A Schmitz W Kneitz S. The transcription factor NRF2 enhances melanoma Malignancy by blocking differentiation and inducing COX2 expression. Oncogene. (2020) 39:6841–55. doi: 10.1038/s41388-020-01477-8, PMID: 32978520 PMC7605435

[29] Zuo D Chen Y Zhang X Wang Z Jiang W Tang F. Identification of hub genes and their novel diagnostic and prognostic significance in pancreatic adenocarcinoma. Cancer Biol Med. (2021) 19(7):1029–46. doi: 10.20892/j.issn.2095-3941.2020.0516, PMID: 34403221 PMC9334760

[30] Álvarez E Falqui M Sin L McGrail JP Perdiguero B Coloma R. Unveiling the multifaceted roles of ISG15: from immunomodulation to therapeutic frontiers. Vaccines (Basel). (2024) 12(2):153. doi: 10.3390/vaccines12020153, PMID: 38400136 PMC10891536

[31] Zhao X Wang J Wang Y Zhang M Zhao W Zhang H. Interferon−stimulated gene 15 promotes progression of endometrial carcinoma and weakens antitumor immune response. Oncol Rep. (2022) 47(6):110. doi: 10.3892/or.2022.8321, PMID: 35445736 PMC9073416

[32] Qiu X Hong Y Yang D Xia M Zhu H Li Q. ISG15 as a novel prognostic biomarker for hepatitis B virus-related hepatocellular carcinoma. Int J Clin Exp Med. (2015) 8:17140–50., PMID: 26770308 PMC4694208

[33] Qu T Zhang W Yan C Ren D Wang Y Guo Y. ISG15 targets glycosylated PD-L1 and promotes its degradation to enhance antitumor immune effects in lung adenocarcinoma. J Transl Med. (2023) 21:341. doi: 10.1186/s12967-023-04135-1, PMID: 37217923 PMC10204161

[34] Zhao W Liu YK Li DJ Zhao XW Deng HY Du NY. The role of interferon-stimulated gene 15 in the occurence and progression of cervical squamous cell carcinoma. J Physiol Pharmacol. (2023) 74(1):77–83. doi: 10.26402/jpp.2023.1.08, PMID: 37245235

[35] Hong G Li H Li M Zheng W Li J Chi M. A simple way to detect disease-associated cellular molecular alterations from mixed-cell blood samples. Brief Bioinform. (2018) 19:613–21. doi: 10.1093/bib/bbx009, PMID: 28200092

[36] Chomczynski P Sacchi N. Single-step method of RNA isolation by acid guanidinium thiocyanate-phenol-chloroform extraction. Anal Biochem. (1987) 162:156–9. doi: 10.1016/0003-2697(87)90021-2 2440339

[37] Pfaffl MW. Quantification strategies in real-time PCR. In: Bustin SA, editor. A-Z of quantitative PCR International University Line (IUL) (2004) p: 87 –112.

[38] Bustin SA. Improving the quality of quantitative polymerase chain reaction experiments: 15 years of MIQE. Mol Aspects Med. (2024) 96:101249. doi: 10.1016/j.mam.2024.101249, PMID: 38290180

[39] Aaronson NK Ahmedzai S Bergman B Bullinger M Cull A Duez NJ. The European Organization for Research and Treatment of Cancer QLQ-C30: a quality-of-life instrument for use in international clinical trials in oncology. J Natl Cancer Inst. (1993) 85:365–76. doi: 10.1093/jnci/85.5.365, PMID: 8433390

[40] Blazeby JM Currie E Zee BC Chie WC Poon RT Garden OJ. Development of a questionnaire module to supplement the EORTC QLQ-C30 to assess quality of life in patients with hepatocellular carcinoma, the EORTC QLQ-HCC18. Eur J Cancer. (2004) 40:2439–44. doi: 10.1016/j.ejca.2004.06.033, PMID: 15519517

[41] Yeo W Mo FK Koh J Chan AT Leung T Hui P. Quality of life is predictive of survival in patients with unresectable hepatocellular carcinoma. Ann Oncol. (2006) 17:1083–9. doi: 10.1093/annonc/mdl065, PMID: 16600982

[42] Bruix J Sherman M Llovet JM Beaugrand M Lencioni R Burroughs AK. Clinical management of hepatocellular carcinoma. Conclusions of the Barcelona-2000 EASL conference. Eur Assoc Study Liver. J Hepatol. (2001) 35:421–30. doi: 10.1016/S0168-8278(01)00130-1, PMID: 11592607

[43] Snell LM McGaha TL Brooks DG. Type I interferon in chronic virus infection and cancer. Trends Immunol. (2017) 38:542–57. doi: 10.1016/j.it.2017.05.005, PMID: 28579323 PMC8059441

[44] Yu R Zhu B Chen D. Type I interferon-mediated tumor immunity and its role in immunotherapy. Cell Mol Life Sci. (2022) 79:191. doi: 10.1007/s00018-022-04219-z, PMID: 35292881 PMC8924142

[45] Fuertes MB Woo SR Burnett B Fu YX Gajewski TF. Type I interferon response and innate immune sensing of cancer. Trends Immunol. (2013) 34:67–73. doi: 10.1016/j.it.2012.10.004, PMID: 23122052 PMC3565059

[46] Ramos EJ Suzuki S Marks D Inui A Asakawa A Meguid MM. Cancer anorexia-cachexia syndrome: cytokines and neuropeptides. Curr Opin Clin Nutr Metab Care. (2004) 7:427–34. doi: 10.1097/01.mco.0000134363.53782.cb, PMID: 15192446

[47] Kirkwood JM Bender C Agarwala S Tarhini A Shipe-Spotloe J Smelko B. Mechanisms and management of toxicities associated with high-dose interferon alfa-2b therapy. J Clin Oncol. (2002) 20:3703–18. doi: 10.1200/JCO.2002.03.052, PMID: 12202672

[48] Abou-Alfa GK Lau G Kudo M Chan SL Kelley RK Furuse J. Tremelimumab plus durvalumab in unresectable hepatocellular carcinoma. NEJM Evid. (2022) 1:EVIDoa2100070. doi: 10.1056/EVIDoa2100070, PMID: 38319892

[49] Cheng AL Qin S Ikeda M Galle PR Ducreux M Kim TY. Updated efficacy and safety data from IMbrave150: Atezolizumab plus bevacizumab vs. sorafenib for unresectable hepatocellular carcinoma. J Hepatol. (2022) 76:862–73. doi: 10.1016/j.jhep.2021.11.030, PMID: 34902530

[50] Yau T Park JW Finn RS Cheng AL Mathurin P Edeline J. Nivolumab versus sorafenib in advanced hepatocellular carcinoma (CheckMate 459): a randomised, multicentre, open-label, phase 3 trial. Lancet Oncol. (2022) 23:77–90. doi: 10.1016/S1470-2045(21)00604-5, PMID: 34914889

[51] Yau T Kang YK Kim TY El-Khoueiry AB Santoro A Sangro B. Efficacy and safety of nivolumab plus ipilimumab in patients with advanced hepatocellular carcinoma previously treated with sorafenib: the checkMate 040 randomized clinical trial. JAMA Oncol. (2020) 6:e204564. doi: 10.1001/jamaoncol.2020.4564, PMID: 33001135 PMC7530824

[52] Verset G Borbath I Karwal M Verslype C Van Vlierberghe H Kardosh A. Pembrolizumab monotherapy for previously untreated advanced hepatocellular carcinoma: data from the open-label, phase II KEYNOTE-224 trial. Clin Cancer Res. (2022) 28:2547–54. doi: 10.1158/1078-0432.CCR-21-3807, PMID: 35421228 PMC9784157

[53] Qin S Chan SL Gu S Bai Y Ren Z Lin X. Camrelizumab plus rivoceranib versus sorafenib as first-line therapy for unresectable hepatocellular carcinoma (CARES-310): a randomised, open-label, international phase 3 study. Lancet. (2023) 402:1133–46. doi: 10.1016/S0140-6736(23)00961-3, PMID: 37499670

[54] Wood LM Pan ZK Seavey MM Muthukumaran G Paterson Y. The ubiquitin-like protein, ISG15, is a novel tumor-associated antigen for cancer immunotherapy. Cancer Immunol Immunother. (2012) 61:689–700. doi: 10.1007/s00262-011-1129-9, PMID: 22057675 PMC4561532

[55] Nguyen HM Gaikwad S Oladejo M Paulishak W Wood LM. Targeting ubiquitin-like protein, ISG15, as a novel tumor associated antigen in colorectal cancer. Cancers (Basel). (2023) 15(4):1237. doi: 10.3390/cancers15041237, PMID: 36831577 PMC9954464

[56] Burks J Fleury A Livingston S Smith JP. ISG15 pathway knockdown reverses pancreatic cancer cell transformation and decreases murine pancreatic tumor growth via downregulation of PDL-1 expression. Cancer Immunol Immunother. (2019) 68:2029–39. doi: 10.1007/s00262-019-02422-9, PMID: 31709456 PMC9886270

[57] Burks J Reed RE Desai SD. Free ISG15 triggers an antitumor immune response against breast cancer: a new perspective. Oncotarget. (2015) 6:7221–31. doi: 10.18632/oncotarget.3372, PMID: 25749047 PMC4466680

[58] Bolado-Carrancio A Lee M Ewing A Muir M Macleod KG Gallagher WM. ISGylation drives basal breast tumour progression by promoting EGFR recycling and Akt signalling. Oncogene. (2021) 40:6235–47. doi: 10.1038/s41388-021-02017-8, PMID: 34556814 PMC8566238

[59] Hirasawa Y Kubota Y Mura E Suzuki R Tsurui T Iriguchi N. Maximum efficacy of immune checkpoint inhibitors occurs in esophageal cancer patients with a low neutrophil-to-lymphocyte ratio and good performance status prior to treatment. Anticancer Res. (2024) 44:3397–407. doi: 10.21873/anticanres.17160, PMID: 39060084

