# Supplementary material for: ISG15 mRNA transcript level in circulating leucocytes prognostic of overall survival in hepatocellular carcinoma patients and correlated with quality of life disturbances involved in anorexia-cachexia
Source: Front Oncol. 2025 Aug 27;15:1589053. doi: 10.3389/fonc.2025.1589053 (PMC12420207; doi:10.3389/fonc.2025.1589053)
Supplement: Supplementary file 1 [file Table1.docx]

**Supplementary Table 1: Quality of Life Tools used in the Study**

| The European Organization for Research and Treatment (EORTC) QLQ-C30 | A 30-item questionnaire for measuring heath related quality of life in cancer patients. This core questionnaire adopts a modular approach to health-related quality of life assessment and can be supplemented by other specific modules. |
| --- | --- |
| EORTC QLQ-HCC18 | The EORTC quality of life group hepatocellular carcinoma-specific module aims to assess all major dimensions of health-related quality of life in patients with hepatocellular carcinoma |
| C30 index score# | $\sum[(100-Physical functioning), (100-Role functioning), (100-Emotional functioning), (100-Cognitive functioning), (100-Social functioning), (100-global health status/QoL), Fatigue, Nausea and vomiting, Pain, Dyspnoea, Insomnia, Appetite loss, Constipation, Diarrhoea, Financial Diffculties]\div15$ |
|  | Score range: 0-100; a higher C30 index score represents a poorer health-related quality of life |
| HCC18 index score# | $\sum(Fatigue. Body Image, Jaundice, Nutrition, Pain, Fever, Abdominal swelling, Sex life)$ $\div8$ |
|  | Score range: 0-100; a higher HCC18 index score represents a poorer health-related quality of life |

#C30 index score and HCC18 index score were computed from EORTC QLQ-C30 and QLQ-HCC18 factor-scores respectively

Legends: EORTC - The European Organization for Research and Treatment

**Supplementary Tables 2 (i)-(iv): Multivariate Logistic Regressions between *ISG15* Gene Expression and Quality of Life Factors Adjusting for Clinical Variables**

| (i) EORTC QLQ-C30 | | | | |
| --- | --- | --- | --- | --- |
|  | Logistic regression | | | |
|  | Odds ratio | 95% confidence intervals | | p-value |
| QLQ-C30 Appetite loss | 1.479 | 1.034 | 2.115 | 0.032 |
|  |  |  |  |  |
| (ii) C30 index score | | | | |
| No factor was identified | | | | |
|  | | | | |
| (iii) EORTC QLQ-HCC18 | | | | |
|  | Logistic regression | | | |
|  | Odds ratio | 95% confidence intervals | | p-value |
| QLQ-HCC18 Nutrition | 1.831 | 1.03 | 3.255 | 0.0385 |
|  |  |  |  |  |
| (iv) HCC18 index score | | | | |
| No factor was identified | | | | |

Legends: EORTC – European Organization for Research and Treatment; QOL - Quality of Life;

*ISG15* – Interferon Stimulated Gene 15
